# Real-World Data from a Multi-Center Study: Insights to Psoriatic Arthritis Care

**DOI:** 10.3390/jcm10184106

**Published:** 2021-09-11

**Authors:** Bogdan Batko, Eugeniusz Kucharz, Marcin Stajszczyk, Marek Brzosko, Włodzimierz Samborski, Zbigniew Żuber

**Affiliations:** 1 Department of Rheumatology and Immunology, Faculty of Medicine and Health Sciences, Andrzej Frycz Modrzewski University, 30-705 Krakow, Poland; 2 Department of Internal Medicine, Rheumatology and Clinical Immunology, Medical University of Silesia, 40-752 Katowice, Poland; ejkucharz@poczta.onet.pl; 3 Silesian Rheumatology Center, Rheumatology and Autoimmune Diseases Department, 43-450 Ustron, Poland; marcins@mp.pl; 4 Department of Rheumatology, Internal Diseases, Geriatrics and Clinical Immunology, Pomeranian Medical University in Szczecin, 70-204 Szczecin, Poland; brzoskom@pum.edu.pl; 5 Department of Rheumatology and Rehabilitation, Poznan University of Medical Sciences, Fredry 10, 61-701 Poznan, Poland; samborskiw@tlen.pl; 6 Department of Pediatrics, Faculty of Medicine and Health Sciences, Andrzej Frycz Modrzewski Krakow University, 30-705 Krakow, Poland; 7 Ward for Older Children with Neurology and Rheumatology Subdivision, St. Louis Regional Specialised Children’s Hospital, 31-503 Krakow, Poland; zbyszekzuber@interia.pl

**Keywords:** treat-to-target, psoriatic arthritis, barriers, real world, difficult-to-treat

## Abstract

Introduction: Real-world data indicate disparities in biologic access across Europe. Objectives: To describe the national structure of PsA care in Poland, with a particular focus on the population of inadequate responders (IRs) and difficulties associated with biologic therapy access. Methods: A pool of rheumatologic and dermatologic care centers was created based on National Health Fund contract lists (*n* = 841), from which 29 rheumatologic and 10 dermatologic centers were sampled randomly and successfully met the inclusion criterium. Additionally, 33 tertiary care centers were recruited. For successful center recruitment, one provider had to recruit at least one patient that met the criteria for one of the four pre-defined clinical subgroups, in which all patients had to have active PsA and IR status to at least 2 conventional synthetic disease-modifying drugs (csDMARDs). Self-assessment questionnaires were distributed among physicians and their patients. Results: Barriers to biologic DMARD (bDMARD) treatment are complex and include stringency of reimbursement criteria, health care system, logistic/organizational, and personal choice factors. For patients who are currently bDMARD users, the median waiting time from the visit, at which the reimbursement procedure was initiated, to the first day of bDMARD admission was 9 weeks (range 2–212; 32% < 4 weeks, 29% 5–12 weeks, 26% 13–28 weeks, 13% with >28 weeks delay). Out of all inadequate responder groups, bDMARD users are the only group with “good” therapeutic situation and satisfaction with therapy. Patient satisfaction with therapy is not always concordant with physician assessment of therapeutic status. Conclusions: Despite the fact that over a decade has passed since the introduction of biologic agents, in medium welfare countries such as Poland, considerable healthcare system barriers to biologic access are present. Out of different IR populations, patient satisfaction with treatment is often discordant with physician assessment of disease status.

## 1. Introduction

Psoriatic arthritis (PsA) is a chronic autoimmune disease that inflicts a significant socioeconomic burden [1]. Dactylitis, spondylitis, peripheral arthritis, and enthesitis are some of the key musculoskeletal manifestations in PsA. Patient-centered care and the importance of psychosocial domains are increasingly recognized in the management strategy of PsA [2]. This paradigm stems from the observations that, on an individual level, the impact of disease extends beyond the skin and joint [3,4,5,6,7,8,9]. It has been shown that achieving minimal disease activity in PsA leads to better quality of life and productivity [10]. However, data indicate that there is a significant degree of undertreatment or no treatment for PsA [11]. A comprehensive overview of the available recommendations from different expert societies is available elsewhere. Patients with psoriasis and PsA frequently report health-risk concerns and dissatisfaction with medication. A substantial proportion have never been informed about systemic treatment by their physician, though this observation is more commonly reported for PsA patients [12]. It has been shown that a shared decision making model of care may lead to improved satisfaction and treatment adherence, at the same time leading to reduction in associated costs [13]. The psoriasis treatment armamentarium is very diverse—arthritic patients may particularly benefit from oral therapies with conventional synthetic disease-modifying antirheumatic drugs (csDMARDs). Methotrexate, sulfasalazine, cyclosporine, leflunomide, and apremilast are all well-established agents. Tumor necrosis factor (TNF) inhibitors are a well-established, safe, and efficacious line of therapy in inflammatory arthritis, which leads to the abrogation of the inflammatory cascade [14]. Other choices in more severe diseases include IL-12/23, IL-17A, or JAK/STAT inhibitors. There is also a wide array of symptomatic therapies, e.g., non-steroid anti-inflammatory drugs (NSAIDs), glucocorticoids (GCs), and non-pharmacological interventions that can aid in disease control. Therapy selection is also guided by the main manifestations of disease and co-occurring diseases (e.g., cardiometabolic comorbidity) [15,16].

The aim of the present study was to provide a structural overview on psoriatic arthritis care in the ambulatory and tertiary care setting. Furthermore, this investigation aimed to describe the experience of patients and providers in the real world, with a focus on perceived barriers and difficulties to biologic care. Four clinical groups with inadequate response were analyzed to evaluate satisfaction, tolerability, and treatment-related concerns.

## 2. Materials and Methods

### 2.1. Center, Provider, and Patient Recruitment

In order to obtain a sample of routine care centers that reflect the structure of ambulatory psoriasis and PsA care in Poland, contract records were retrieved from the National Health Fund to create a center recruitment pool. Two analogous studies were conducted for psoriasis and psoriatic arthritis, each with different inclusion criteria for patient populations, but for the same center recruitment pool. These studies were non-interventional in design. There was no requirement nor guidance for the current treatment strategy, except for the inclusion criterium of prior failure of 2 csDMARDs (more details in Table 1). Centers holding a contract for the treatment of rheumatic and dermatologic conditions in the ambulatory setting (only) were analyzed based on the available records for 2017. Data ordering and center lists were created based on contract value, and a center recruitment pool that comprised over half of total expenditures in both fields was created. This step was performed to ensure that centers with actively practicing specialists were invited to participate. This is because centers that are allocated greater funds are more likely to consistently provide care for a greater number of rheumatic patients, thus being able to successfully recruit PsA patients who fulfill the selective criteria for patient-level analysis.

The investigators aimed to include centers that could recruit the target patient groups. Initially, providers were queried about their ability to recruit patients that fall into the clinical subgroups, as depicted in Table 1. Recruitment of specialists was based on declarative consent and intent to participate in the study. Physicians had to confirm that they admit at least one patient from the pre-defined patient populations that are of interest to this study. Providers who participated in the study provided estimates regarding the number of patients (from the aforementioned groups) that are admitted in the course of an average month of practice. Based on analyses for each clinical subgroup of interest, patient quota from a given segment was allocated to a physician based on the estimates they provided previously. The specialists invited consecutive patients to participate in the study if they fulfilled the inclusion criteria. Each center was included in final analyses only if they supplied data (inclusion criterium) from one physician who was successful in the recruitment of at least one consecutive patient that fulfilled the pre-defined patient subgroup criteria. Medical data (SAQ) were gathered during an outpatient visit. A minimum sample of 30 patients from each clinical subgroup of interest was pre-defined, but could be exceeded, and the final clinical sample counted a total of 150 patients (36 patients each in both segments A and B, 39 patients each in both segments C and D). We did not enforce any assessment of composite indices or more objective measures of inflammatory activity to validate the criterium of active PsA, the judgment of which was at the physician’s discretion. In part, this was guided by prior observations in rheumatoid arthritis, which showed low uptake of composite indices and clinical examination as the major decision tool in practice [17]. Furthermore, we did not specifically aim to compare treatment effectiveness nor perform comparative analyses regarding therapeutic schemes. This investigation was focused on the patient and provider perspectives, as gathered using tailored questionnaires (each patient group received a specific questionnaire, with some overlap regarding clinical characteristics). The inclusion criteria on a patient-level are based on the validity of provider claims, which is a major limitation. 

Recruitment of centers based on public payer records is likely to reflect the nationwide structure of care in Poland due to the unique character of our health system. The vast majority of healthcare services (specifically, the health sector, which is funded by the public) in Poland is constitutionally equal (Article 68), regardless of material situation, to all citizens. Sagan et al. have provided a comprehensive review on Polish health care, reporting that 98% of the population is covered by compulsory health insurance, which enables a broad range of services, while voluntary health insurance plays a minor role [18]. The assumption the investigators agreed on is that analysis of reimbursement contracts, which are based on center characteristics and patient populations, is likely to reflect the routine circumstances of dermatology and rheumatology on a national level. 

**Figure 1:**
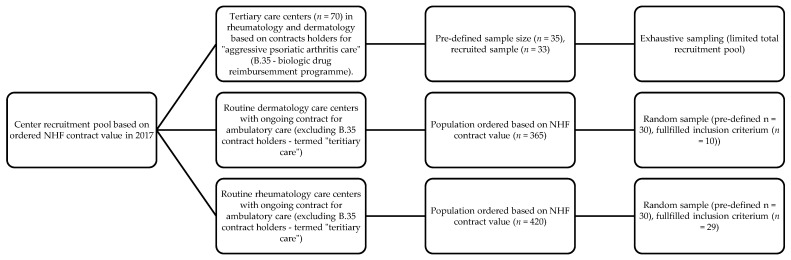
Process of recruiting rheumatology and dermatology care centers into the study.

In total, 29 rheumatology and 10 dermatology care centers were randomly sampled from the aforementioned Health Fund center pool (see Figure 1). If a center was not qualified (i.e., if the physician representing this center did not recruit a patient fulfilling the inclusion criterium of active psoriatic arthritis and had inadequate response to at least to 2 csDMARDs, and further did not qualify into any of the groups described in Table 1), recruitment of this center was considered as failed. If a provider failed in the recruitment process, additional sampling of a different, random center was performed according to the regional geographical distribution. The pre-set dermatology and rheumatology center sample counts were not fulfilled, which was due to a difficulty in recruiting the patient population of interest in centers that provide only ambulatory care (see Figure 1; the center recruitment pools for dermatology and rheumatology excluded centers with B.35 contracts, which are usually allocated to tertiary care). This is likely to reflect the uncommon character of these disease phenotypes in the outpatient setting (particularly for active PsA in dermatology), which would likely merit referral to a rheumatologist earlier in the disease course.

Additionally, a sample of specialized rheumatology centers that hold contracts for biologic therapy was recruited using exhaustive sampling (further termed “tertiary care”). Random sampling was not performed in the latter case due to a limited recruitment pool (*n* = 70). In total, the study was performed on a sample of 72 physicians from each of the recruited centers. Due to an over-representation of biologic care centers, population weights were assigned during analyses on a center-level.

### 2.2. Data Gathering and Processing

Self-assessment questionnaires (SAQ) were physically distributed to both physicians and patients alike (patients and providers had to complete different sections of the questionnaire, and questionnaires for clinical subgroups had different questions respective to the clinical context). Supplementation of survey data was performed with a computer-assisted web interview (CAWI) tool distributed among providers. Data were collected between November 2017 and May 2018.

### 2.3. Statistical Analysis

Patient counts and percentages are reported for categories, and mean (SD) or median IQR/range is reported for continuous variables, in accordance with sample distribution, which was evaluated using the Shapiro–Wilk test. Categorical data were analyzed with the ×2 test or Fisher exact test, as appropriate. Comparison across groups was performed with Mann–Whitney U test or t-test, according to distribution. A P-value of less than 0.05 was set as significant, and the tests were two-tailed. Statistical analyses were performed using SPSS software ver. 27.0 (IBM, Armonk, NY, USA), and MS Excel (Microsoft, Redmond, Washington, USA).

## 3. Results

### 3.1. Center and Physician-Level Estimates for the Population of Patients with Active Psoriatic Arthritis, including Inadequate-Responders and Patients Who Have Never Been Treated Systemically 

In the total center sample (*n* = 841), 48%, 43%, and 8% of the centers provide dermatologic, rheumatologic, and tertiary care, respectively (details in Figure S1). Due to the observed differences, for center-level data, population weighing was adopted to account for the participation of particular centers. 

Centers for psoriatic arthritis care provide treatment for a median of 40 (range 1–400) patients with active PsA. Of these patients, a median of 5 (range 0–65) has never been treated systemically, while a median of 10 (range 0–150) had an inadequate response to at least two csDMARDs (further termed “inadequate responders” or “IRs”).

On a provider level (*n* = 72, one physician from each of the recruited centers), specialists provide care for a median of 30 (range 1–200) patients with active PsA. Providers report that a median of 1 (range 0–30) individuals have never been treated with systemic agents, while 15 (range 1–88) are considered IRs.

“Please estimate how many adult patients, suffering from active psoriatic arthritis, are currently under [your care/the care of your center]”

“Please estimate how many adult patients, suffering from active psoriatic arthritis, currently under [your care/the care of your center], [have inadequate response to at least 2 csDMARDs/have not been treated with systemic agents to date]?”

### 3.2. Patient-Reported Difficulties Related to the Drug Reimbursement Procedure 

Based on group A patients’ responses (*n* = 31), the median waiting time from the visit at which the reimbursement procedure was initiated to the first day of bDMARD admission was 9 weeks (range 2–212; 32% <4 weeks, 29% 5–12 weeks, 26% 13–28 weeks, 13% with >28 weeks delay). The median center waiting time (complete data for *n* = 16) to tertiary care (i.e., the time from patient referral to visit at the bDMARD-treating center) was 2 months (<1 month for 38% of patients, 1 to 6 months for 50%, >6 months for 12%).

The three most common difficulties related to the drug program procedure (as reported by group A patients (*n* = 36)) were the uncertainty over the administrative decision (69%), overall amount of time allocated (58%), and requirement of documented prior failure of 2 csDMARDs (53%). It was observed that 47% of patients spend less than 3 h at the treatment center, 39% require several hours, while 14% need to allocate a whole day; 19% of patients require the aid of a companion, and only 17% report no need for work leave in order to receive the treatment (of note, 33% are unemployed, and 50% require sick leave).

“How long did you wait to be admitted to the facility to which you were referred to complete formalities related to qualification for biological treatment under the drug program (i.e., how much time passed since the visit at which you received referral)?”

“The period of time from the visit at which the procedure of qualifying a patient for active biological treatment of PsA (under the drug program) was started to the day the patient was given the first dose of a biological drug”

“What was troublesome or problematic for you while applying for biological treatment of PsA? (under the drug program, you can choose more than 1 answer)”

“How long does it usually take for you to visit a facility that provides biological treatment for PsA (as part of a drug program)?”

“In order to be administered the biological drug for PsA (under the drug program), do you ever have to take time off from work?”

“In order to visit a facility that provides biological treatment of PsA (under the drug program), do you ever have to engage the assistance of someone else?”

### 3.3. Availability and Barriers to Biologic Treatment—A Physician’s Perspective

In general, providers (*n* = 72) claimed that when comparing with the previous year, the subset of outpatients with biologic access has increased (56% of providers stated that, within the prior 12 months, the availability of biological treatment has increased, while 38% estimated that it has not changed; the remainder are of the view that accessibility has decreased).

“How do you think the availability of biological treatment for adult patients with active PsA has changed in the last 12 months?”

The three most common barriers to biologics (listed in decreasing order), as per the provider responses (*n* = 72): general inclusion criteria for the drug program (65% of responders); specifically, the stringent requirement of prior failure of treatments (64%); and actual availability of biological drugs (43%) (see full list in Supplementary Material, Section 1.1).

“In your opinion, what are the main barriers to inclusion of adult patients with active PsA into biological therapy (within the drug program)?” [Multiple responses were allowed.]

When asked to grade different factors that influence availability of bDMARD therapy on a 10-point Likert scale, the average physician responses (*n* = 72) were between 6 and 7 (6.6—time limitations for bDMARD program itself; 6.4—administrative procedures; 6.3—program criteria; and 6.2—NHF fund budget). It is worth noting that at least one-third of physicians classified these difficulties between 8 and 10.

“The following list includes selected factors that may affect the availability of biological therapy for patients treated for active PsA. For each of them, please rate how much each factor currently constitutes a barrier to the enrollment of your patients in the biological treatment program.”

Every second physician (*n* = 72) (51%) expressed the view that funds for biologics in PsA are insufficient and they estimated (on average) that close to one-third (31%) of patients miss the opportunity for bDMARD therapy despite clinical eligibility. In total, 64% of physicians (*n* = 72) claimed bureaucratic difficulties and individual physician workload are “very common” (21%) or “rather common” (43%) barriers to drug access during the application process. About one-third of providers estimated that these difficulties are uncommon in practice, while only 3% declared that they do not occur.

“Do you think the funds allocated to the biological treatment program for adult patients with active PsA are sufficient (taking into account the number of outpatient patients eligible for such treatment) or rather insufficient (i.e., treatment is lacking for a proportion of patients who are eligible)?”

“In what percentage of adult patients with active PsA admitted by you (in open treatment, who are eligible for biological treatment under the drug program) is treatment not initiated due to insufficient funding.”

“How often do you think it might occur that providers treating adult patients with active PsA give up the introduction of biological therapy due to bureaucratic difficulties and a significant workload (related to the qualification and monitoring of patients under the drug program)? Please think not only about your own experiences, but also about the situations your colleagues encounter.”

### 3.4. Common Causes for Withdrawal of bDMARD Therapy 

Group B subjects were, by definition, patients with prior history of biological treatment, who did not achieve low disease activity (treatment target) until present day. Among the reasons for stopping biologics, the most common causes reported by patients (*n* = 36) and providers (*n* = 26) were defined as side effects fulfilling criteria defined in the drug program (50%), personal decisions for withdrawal (44%), side effects other than specified within the drug program (6%), and others (8%). However, providers (*n* = 26) are of the view that side effects that do not fulfill drug program exclusion criteria are more common than side effects that meet the drug program exclusion criteria (40% vs. 34%, respectively).

Concerning the significant adverse effects reported by patients (missing data, available sample *n* = 18), serious infections with a severe course (39%), allergic reactions (33%), and neoplastic disease (17%) were the most common factors reported by patients. Physicians provided similar responses (*n* = 16).

Among the justifications for individual decisions of withdrawal (missing data, available sample *n* = 16), planned procreation (31%), longer travel (25%), and frequent infections (25%) were most commonly reported by patients. Similar factors were present in provider responses (*n* = 19) for these patients. 

### 3.5. Patient Satisfaction in Treatment and Physician Assessment of Therapeutic Effectiveness—The Relationship with Current bDMARD Therapy 

In the total sample (*n* = 150), the mean age of patients was 47 years (±13), and the gender distribution was equal (50.7% were female). The majority of patients had longstanding disease, with a median duration of 8 years (IQR 8.25, missing data for *n* = 16). Only 3 (2.2%) patients presented within 2 years of disease onset (i.e., early PsA). 

**Figure 2:**
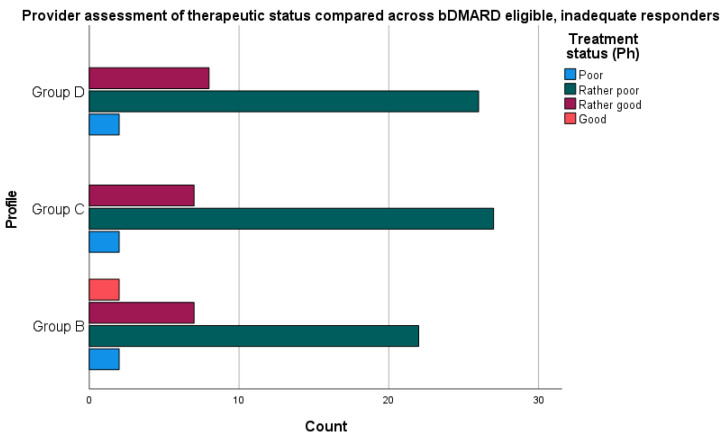
Overview of specialist assessment of therapeutic status according to the question stem, “Assessment of the current therapeutic situation of the patient related to the therapy of active PsA as part of the drug program.” (an additional analysis showed no statistically significant difference between group D and C).

**Figure 3:**
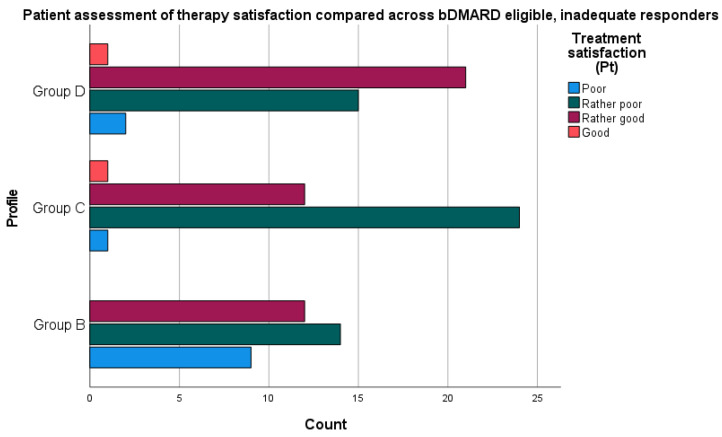
Overview of patient assessment of treatment satisfaction according to the question stem, “Are you satisfied with the treatment currently used, with regard to the lack of biological treatment of PsA under the drug program?”.

“Effective” or “satisfactory” assessment of disease-modifying therapy/clinical status was defined based on responses on 4-point Likert scales (1—“unsatisfied/poor”; 2—“rather unsatisfied/poor”; 3—“rather satisfied/good”; and 4—“satisfied/good”). When excluding patients with biologic therapy, the remaining clinical groups have an overall “rather poor” therapeutic situation (see Figure 2). This contrasts with treatment satisfaction in these groups (see Figure 3).

When examining the overall sample of inadequate responders with PsA, the McNemar test determined that there was a significant difference in disease evaluation when matching patient satisfaction to physician evaluation of therapy (complete data in *n* = 139, *p =* 0.001).

We also examined the proportion of discordance between patient and provider-level assessments based on the concordance of “satisfaction” and “therapeutic status” scores with regard to disease-modifying treatment. Comparing across all patient profiles, the proportions of discordance (defined as binary variable of whether “satisfaction” and “therapeutic situation” were equivalent on a 4-point scale) were not statistically significantly different. 

### 3.6. Comparison of bDMARD Users and Patients Who Qualify, Are Eligible, but Chose Not to Initiate Biologic Therapy

We further examined the difference across PsA patients who are bDMARD users and patients that qualify for therapy according to the drug program (and are clinically eligible according to the physician), but do not choose to initiate therapy (Table 2). Among patient justifications (*n* = 39) for lack of bDMARD therapy interest, fears over treatment (49%), requirements for visits at tertiary centers for drug admission (33%), overall amount of time allocated (33%), difficulty of the qualification procedure (31%), travel (26%), and the amount of time spent at the center for drug admission (23%) were the most frequent responses (multiple responses were allowed). In the physician sample (*n* = 41, only providers that had at least one patient from the aforementioned groups), difficulty of the qualification procedure (27%), fears over treatment (24%), and requirement for visits (22%) were the most common responses.

Patients from group A and C did not statistically significantly differ in current csDMARD use (methotrexate, sulfasalazine, leflunomide, or cyclosporine), current or prior orthopedic treatment, or adjunct physical rehabilitation. Interestingly, the educational level was a factor that differed between these patient samples. However, the use of NSAIDs was far more common in group C, which suggests that these patients require more intensive symptomatic therapy. 

We further explored this trend in the general sample (see Table 3), and observed that current NSAID use is associated with worse physician assessment of therapeutic status, but not with patient satisfaction. 

## 4. Discussion

This is a multicenter study based on a random sample of rheumatology and dermatology care centers. The aim of this study is to provide a descriptive overview of ambulatory and specialized care for PsA in Poland, using data gathered on a provider and patient level. An analysis of four samples of patients with IRs and active PsA is reported. These groups represent different “clinical phenotypes” for patients who are eligible for bDMARDs. Data were examined with regard to self-reported satisfaction with treatment and physician assessment of therapeutic status. This study also identified barriers and difficulties tied to the drug reimbursement program. Its main strength is a design that gathers data from a wide array of routine care centers with national geographic distribution, while analyses are conducted on multiple levels (center-, physician-, and patient-levels). The main limitation of the presented results is bias associated with claim-based data and the subjective nature of inclusion criteria.

We observed that the population of patients with inadequate response to 2 csDMARDs is heterogenous with regard to patient satisfaction and therapeutic status, as assessed by a specialist. Variable non-medical factors seem to contribute to the therapeutic decisions that a patient who is clinically eligible for bDMARD therapy may undertake, which may translate into a worse disease status. A comparison of current biologic users with bDMARD eligible patients (group C), who have chosen not to initiate bDMARD therapy, showed that satisfaction on a patient level and provider-level evaluation of treatment efficacy may be significantly worse. Considering the importance of treat-to-target [19] strategies in rheumatology, the present guidelines from different expert societies recommend inflammatory control, though the target is at the discretion of the physician. However, it has been reported that patients may not be inclined to such an approach due to the need for more frequent visits, consideration of costs, and fears over side effects [15,16,20]. In our study, in group C patients, fears over bDMARDs and the complex difficulties associated with the drug reimbursement procedure were reported as major barriers to the drug program. These individuals did not initiate therapy, and have worse treatment satisfaction and poor disease status when compared with bDMARD users, despite the treatment being available. Furthermore, the proportion of patients who require NSAID use in group C is nearly twofold compared with those in group A, which indicates the need for symptomatic relief. An analysis of the overall sample of inadequate responders suggests that NSAID use may be tied to poor therapeutic status, but not to patient satisfaction. It should be noted that although there is no definite guidance regarding NSAID use, this observation may indicate that the current therapeutic strategy is insufficient. These findings are in line with data from multinational studies indicating that pain and fatigue remain significant in a proportion of patients on disease-modifying treatment [21,22]. These findings have several implications for practice. 

We observed that rheumatologists and patients frequently report administrative and financial difficulties among the main barriers to biologic therapy. This finding is particularly concerning as over a decade has passed since the advent of biological drugs in clinical practice, and these staple agents in the rheumatologist armamentarium appear to be restricted. The observation that the time from initiation of drug reimbursement procedures to actual drug admission may be last 3 months or more is striking. Earlier reports have shown that alongside other Eastern European countries, Poland has a particularly low uptake of biologics [23]. Vast disparities in DMARD access (similarly, mainly financial and administrative) appear to be prevalent across Europe [24]. 

Our study shows that there is variability in the population of inadequate responders with active PsA. Patients experience a number of difficulties; attaining access to biologics, despite eligibility, maintaining access following drug program withdrawal, and even in applying for the program due to the troublesome nature of the procedure. For a proportion of patients, associated costs and logistic difficulties are an important barrier to bDMARD initiation. Economic implications of a given treatment strategy are, unfortunately, a major challenge in routine practice. Analyses based on healthcare systems with commercial insurance show that aside from improvements in daily activity, drug costs, route, and frequency of administration are ranked among the most important characteristics of medication [25]. Direct expenditure is, however, only one constituent of the socioeconomic burden of disease, with indirect costs accounting for approximately 50 to 70% of total costs in PsA [1]. In our study, indirect costs are reflected in the responses of patients who report the need for transportation, assistance from others, time allocation, and work leave, which enable the admission of the biologic agent in a tertiary care center. In the present study, the time delays in initiation of therapy tied to the administrative procedures are considerable. Whether this translates to long-term consequences is probable, but requires further confirmation. This lasts until a patient fulfills stringent drug program criteria or qualifies for a clinical trial, which potentially leads to a greater degree of disability and impaired productivity. Although these findings cannot be directly generalized to other healthcare systems, and the validity of claim-based data needs to be confirmed, the reporting of these difficulties from the perspective of a medium welfare country is important. These observations indicate a need for specialists in rheumatology and dermatology to raise awareness among policymakers in the healthcare sector. While novel pharmaceuticals (e.g., Th17-IL-17 axis or small-molecule Janus kinase inhibitors) are currently slowly introduced into the market, a significant proportion of patients in medium-to-lower welfare countries still remain with limited access to “older” drug generations. This begs the question of whether the introduction of novel pharmaceuticals has a significant impact on the real-world population of patients across lower welfare nations.

In Scandinavian PsA populations, responder satisfaction with biologics was estimated at close to 60% [12]. Prior reports based on national survey data in the United States suggest that about 45% of PsA patients may be dissatisfied with their treatment [11]. It appears that patients are dissatisfied with particular domains of disease, while they are accepting of others. Data from a variety of autoimmune diseases suggest that subjects using intravenous biologics show high satisfaction with medication and have positive views over the interaction with physicians at infusion facilities [26]. Our data indicate that PsA patients widely recognize negative logistic and time-related aspects of tertiary care visits. However, it seems that Polish patients who are bDMARD users are satisfied with their disease status, despite responses about the inconvenience of visits and difficulties in attaining access. Disparities between clinical groups in our study show that treatment satisfaction in PsA is nuanced. The attitude to treatment can be different on a geographical level and may be dependent on healthcare. The present findings indicate that despite biologic eligibility, patients without access to biologics and suffering from active, unresponsive disease still view the treatment process as satisfactory (in some cases). This contrasts with provider perspectives on disease control as “poor”. Some studies have shown that higher disease activity is linked to residence in countries with low wealth [27]. Patients from high welfare countries may experience greater symptom burden, which may be explained as different coping strategies [28]. PsA patients in Poland may be satisfied with their treatment despite poor clinical status due to sociocultural differences and perception of disease. However, this hypothesis requires confirmation in future studies.

Tveit et al. recently examined patient experience with treatment in Scandinavian populations. Among the most common reasons for dissatisfaction with medications, side-effects, uncertainty over effectiveness, limitations to daily activities, and need for self-injections were prominent [12]. Multinational data indicate that patients with psoriasis and PsA frequently discontinue therapy (45% for biologics), often due to safety issues or low effectiveness [29]. In our sample, patients withdrew from therapy due to a variety of personal causes (aside from drug program exclusion criteria), but side effects were an important factor identified by physicians (of note, providers reported that side effects that do not fulfill the program criteria are the major culprit). Reports indicate that patients prioritize biologic safety, followed by efficacy. It should be noted that some studies indicate that, in up to 40% of cases, treatment preferences stand to benefit from communication with providers [30]. One of the positive findings of our study is that nearly all patients experienced discussion over second-line treatment (i.e., biologics) with their providers. This finding suggests that the difficulties in attaining biologic access are not a question of patient-provider knowledge, but rather impediments on an organizational and legislative level.

Several limitations due to methodology are apparent in this study. The design for data collection was based on in-person questionnaires, which are prone to recall and misattribution bias. The validity of these claim-based data is the major limitation of this study. We sought to limit the degree of bias by conducting an investigation on both a patient and a provider level (with separate survey elements for patients and physicians), in addition to supplementation by an electronic questionnaire for the physician. We did not gather in-depth clinical data or more objective measures of disease activity, such as composite indices, as this was not within the scope and resources of the investigators.

## 5. Conclusions

This is a multicenter study that collected data on a patient and provider level to provide a descriptive overview on the population of inadequate responders to at least 2 csDMARDS, with specialist assessment of active psoriatic arthritis and clinical eligibility for bDMARD therapy. Due to a variety of nonmedical factors, such as administrative barriers regulating the drug program reimbursement criteria, or personal factors such as patient fears over side effects, patients do not achieve access to biologic treatment. In our sample of patients, compared with the remaining IR groups, bDMARD users were the only population with uniformly “good” therapeutic situation and “good” treatment satisfaction. The remaining clinical subgroups most often have a “rather poor” disease status. Further research is necessary to validate these findings and elucidate what intervention will enable patients to overcome non-medical barriers to bDMARDs.

## Figures and Tables

**Table 1:** Criteria for recruitment into clinical subgroups of interest.

Inclusion Criteria for Each Patient Group	A	B	C	D
Active psoriatic arthritis according to specialist	+	+	+	+
Inadequate response to at least 2 csDMARDs	+	+	+	+
Ongoing biologic treatment	+	−	−	−
Prior biologic treatment without achievement of at least low-disease activity up to present day	−	+	−	−
Fulfilling biologic reimbursement criteria	+	+	+	−
Biologic therapy eligible under specialist assessment	+	+	+	+

**Table 2:** Demographic and clinical characteristics of group A (current bDMARD users) and C (bDMARD drug program eligible; did not initiate) patients among the population of inadequate responders with active PsA.

Variable		Group A	Group C	*p*-Value
Age in years, Mean (SD) (*n* = 68)		44.96 (11.3)	46.90 (11.9)	0.464
Gender, female (%) (*n* = 75)		15 (44.1)	19 (55.9)	0.540
Duration of PsA in years, Median (IQR) (*n* = 68)		10 (8)	7.5 (6)	0.088
Education level, count (%) (*n* = 75)	High	17 (73.9)	6 (26.1)	0.007
	Moderate	18 (38.3)	29 (61.7)	
	Basic	1 (20)	4 (80)	
Residence, count (%) (*n* = 75)	Rural or small city	23 (46.4)	24 (53.6)	0.833
	Large city	13 (48.9)	15 (51.1)	
Employed, count (%) (*n* = 74)	Yes	25 (46.3)	29 (53.7)	0.777
	No	10 (50)	10 (50)	
High (above average PL salary) income, count (%) (*n* = 75)	Approx. >1300 USD	11 (64.7)	6 (35.3)	0.117
	Approx. <1300 USD	25 (43.1)	33 (56.9)	
Patient satisfaction with treatment, count (%) (*n* = 74)	Dissatisfied	0 (0)	25 (100)	<0.001
	Satisfied	36 (73.5)	13 (26.5)	
Provider assessment of therapeutic status, count (%) (*n* = 70)	Poor	0 (0)	29 (100)	<0.001
	Good	34 (82.9)	7 (17.1)	
NSAIDs currently (*n* = 63)	Yes	16 (36.4)	28 (63.6)	0.019
	No	13 (68.4)	6 (31.6)	

**Table 3:** The relationship between NSAID use and provider assessed therapy efficacy/patient-reported treatment satisfaction in the total patient sample.

NSAIDs Currently	Therapeutic Status (Provider)			Patient Treatment Satisfaction		
	Good	Poor	*p*-Value	Good	Poor	*p*-Value
Yes	34 (35.4%)	62 (64.6%)	0.010	54 (52.4%)	49 (47.6%)	0.464
No	19 (63.3%)	11 (36.7%)		18 (60%)	12 (40%)	

## Data Availability

Any specific questions or data queries are available from the Authors upon request.

## Supplementary Materials

The following are available online at https://www.mdpi.com/article/10.3390/jcm10184106/s1, Figure S1: Representation of centers recruited in the sample and the respective proportions compared to the center recruitment pool. Due to the disparities, center-level data had to be weighed against the population average.

## References

[1] Lee S., Mendelsohn A., Sarnes E. (2010). The Burden of Psoriatic Arthritis: A Literature Review from a Global Health Systems Perspective. Pharm. Ther..

[2] Batko B. (2020). Patient-Centered Care in Psoriatic Arthritis-A Perspective on Inflammation, Disease Activity, and Psychosocial Factors. J. Clin. Med..

[3] Betteridge N., Boehncke W.-H., Bundy C., Gossec L., Gratacós J., Augustin M. (2016). Promoting Patient-Centred Care in Psoriatic Arthritis: A Multidisciplinary European Perspective on Improving the Patient Experience. J. Eur. Acad. Dermatol. Venereol..

[4] Boehncke W.-H., Menter A. (2013). Burden of Disease: Psoriasis and Psoriatic Arthritis. Am. J. Clin. Dermatol..

[5] Gossec L., de Wit M., Kiltz U., Braun J., Kalyoncu U., Scrivo R., Maccarone M., Carton L., Otsa K., Sooäär I. (2014). A Patient-Derived and Patient-Reported Outcome Measure for Assessing Psoriatic Arthritis: Elaboration and Preliminary Validation of the Psoriatic Arthritis Impact of Disease (PsAID) Questionnaire, a 13-Country EULAR Initiative. Ann. Rheum. Dis..

[6] Reich K., Krüger K., Mössner R., Augustin M. (2009). Epidemiology and Clinical Pattern of Psoriatic Arthritis in Germany: A Prospective Interdisciplinary Epidemiological Study of 1511 Patients with Plaque-Type Psoriasis. Br. J. Dermatol..

[7] Mathieu S., Couderc M., Pereira B., Dubost J.-J., Malochet-Guinamand S., Tournadre A., Soubrier M., Moisset X. (2020). Prevalence of Migraine and Neuropathic Pain in Rheumatic Diseases. J. Clin. Med..

[8] Cometi L., Bruni C., Chiti N., Tofani L., Nacci F., Bartoli F., Bellando-Randone S., Melchiorre D., Fiori G., Guiducci S. (2020). Effect of Dysmetabolisms and Comorbidities on the Efficacy and Safety of Biological Therapy in Chronic Inflammatory Joint Diseases. J. Clin. Med..

[9] Olejniczak-Staruch I., Ciążyńska M., Sobolewska-Sztychny D., Narbutt J., Skibińska M., Lesiak A. (2021). Alterations of the Skin and Gut Microbiome in Psoriasis and Psoriatic Arthritis. Int. J. Mol. Sci..

[10] Coates L.C., Orbai A.-M., Morita A., Benichou O., Kerr L., Adams D.H., Shuler C.L., Birt J., Helliwell P.S. (2018). Achieving Minimal Disease Activity in Psoriatic Arthritis Predicts Meaningful Improvements in Patients’ Health-Related Quality of Life and Productivity. BMC Rheumatol..

[11] Armstrong A.W., Robertson A.D., Wu J., Schupp C., Lebwohl M.G. (2013). Undertreatment, Treatment Trends, and Treatment Dissatisfaction among Patients with Psoriasis and Psoriatic Arthritis in the United States: Findings from the National Psoriasis Foundation Surveys, 2003–2011. JAMA Dermatol..

[12] Tveit K.S., Duvetorp A., Østergaard M., Skov L., Danielsen K., Iversen L., Seifert O. (2019). Treatment Use and Satisfaction among Patients with Psoriasis and Psoriatic Arthritis: Results from the NORdic PAtient Survey of Psoriasis and Psoriatic Arthritis (NORPAPP). J. Eur. Acad. Dermatol. Venereol..

[13] Lofland J.H., Johnson P.T., Ingham M.P., Rosemas S.C., White J.C., Ellis L. (2017). Shared Decision-Making for Biologic Treatment of Autoimmune Disease: Influence on Adherence, Persistence, Satisfaction, and Health Care Costs. Patient Prefer. Adherence.

[14] Jang D., Lee A.-H., Shin H.-Y., Song H.-R., Park J.-H., Kang T.-B., Lee S.-R., Yang S.-H. (2021). The Role of Tumor Necrosis Factor Alpha (TNF-α) in Autoimmune Disease and Current TNF-α Inhibitors in Therapeutics. Int. J. Mol. Sci..

[15] Coates L.C., Kavanaugh A., Mease P.J., Soriano E.R., Laura Acosta-Felquer M., Armstrong A.W., Bautista-Molano W., Boehncke W.-H., Campbell W., Cauli A. (2016). Group for Research and Assessment of Psoriasis and Psoriatic Arthritis 2015 Treatment Recommendations for Psoriatic Arthritis. Arthritis Rheumatol..

[16] Smolen J.S., Braun J., Dougados M., Emery P., Fitzgerald O., Helliwell P., Kavanaugh A., Kvien T.K., Landewé R., Luger T. (2014). Treating Spondyloarthritis, Including Ankylosing Spondylitis and Psoriatic Arthritis, to Target: Recommendations of an International Task Force. Ann. Rheum. Dis..

[17] Batko B., Korkosz M., Juś A., Wiland P. (2019). Management of Rheumatoid Arthritis in Poland–Where Daily Practice Might Not Always Meet Evidence-Based Guidelines. Arch. Med. Sci..

[18] Sagan A., Panteli D., Borkowski W., Dmowski M., Domanski F., Czyzewski M., Gorynski P., Karpacka D., Kiersztyn E., Kowalska I. (2011). Poland Health System Review. Health Syst. Transit..

[19] Coates L.C., Moverley A.R., McParland L., Brown S., Navarro-Coy N., O’Dwyer J.L., Meads D.M., Emery P., Conaghan P.G., Helliwell P.S. (2015). Effect of Tight Control of Inflammation in Early Psoriatic Arthritis (TICOPA): A UK Multicentre, Open-Label, Randomised Controlled Trial. Lancet.

[20] Ogdie A., de Wit M., Callis Duffin K., Campbell W., Chau J., Coates L.C., Eder L., Elmamoun M., FitzGerald O., Gladman D.D. (2017). Defining Outcome Measures for Psoriatic Arthritis: A Report from the GRAPPA-OMERACT Working Group. J. Rheumatol..

[21] Conaghan P.G., Alten R., Deodhar A., Sullivan E., Blackburn S., Tian H., Gandhi K., Jugl S.M., Strand V. (2020). Relationship of Pain and Fatigue with Health-Related Quality of Life and Work in Patients with Psoriatic Arthritis on TNFi: Results of a Multi-National Real-World Study. RMD Open.

[22] Conaghan P.G., Strand V., Alten R., Sullivan E., Blackburn S., Huneault L., Tian H., Gandhi K., Jugl S. (2017). OP0107 Pain Still Remains a High Unmet Need among Psoriatic Arthritis Patients Receiving Existing Biologic Treatment: Results from a MultiNational Real-World Survey. Ann. Rheum. Dis..

[23] Rencz F., Kemény L., Gajdácsi J.Z., Owczarek W., Arenberger P., Tiplica G.S., Stanimirović A., Niewada M., Petrova G., Marinov L.T. (2015). Use of Biologics for Psoriasis in Central and Eastern European Countries. J. Eur. Acad. Dermatol. Venereol..

[24] Naldi L., Cazzaniga S., Di Mercurio M., Grossi E., Addis A. (2017). Psocare study centres Inequalities in Access to Biological Treatments for Psoriasis: Results from the Italian Psocare Registry. Br. J. Dermatol..

[25] Xu Y., Sudharshan L., Hsu M.-A., Koenig A.S., Cappelleri J.C., Liu W.F., Smith T.W., Pasquale M.K. (2018). Patient Preferences Associated with Therapies for Psoriatic Arthritis: A Conjoint Analysis. Am. Health Drug Benefits.

[26] Bolge S.C., Eldridge H.M., Lofland J.H., Ravin C., Hart P.J., Ingham M.P. (2017). Patient Experience with Intravenous Biologic Therapies for Ankylosing Spondylitis, Crohn’s Disease, Psoriatic Arthritis, Psoriasis, Rheumatoid Arthritis, and Ulcerative Colitis. Patient Prefer. Adherence.

[27] Putrik P., Ramiro S., Keszei A.P., Hmamouchi I., Dougados M., Uhlig T., Kvien T.K., Boonen A. (2016). Lower Education and Living in Countries with Lower Wealth Are Associated with Higher Disease Activity in Rheumatoid Arthritis: Results from the Multinational COMORA Study. Ann. Rheum. Dis..

[28] Hifinger M., Putrik P., Ramiro S., Keszei A.P., Hmamouchi I., Dougados M., Gossec L., Boonen A. (2016). In Rheumatoid Arthritis, Country of Residence Has an Important Influence on Fatigue: Results from the Multinational COMORA Study. Rheumatology.

[29] Lebwohl M.G., Bachelez H., Barker J., Girolomoni G., Kavanaugh A., Langley R.G., Paul C.F., Puig L., Reich K., van de Kerkhof P.C.M. (2014). Patient Perspectives in the Management of Psoriasis: Results from the Population-Based Multinational Assessment of Psoriasis and Psoriatic Arthritis Survey. J. Am. Acad. Dermatol..

[30] Aletaha D., Husni M.E., Merola J.F., Ranza R., Bertheussen H., Lippe R., Young P.M., Cappelleri J.C., Brown T.M., Ervin C. (2020). Treatment Mode Preferences in Psoriatic Arthritis: A Qualitative Multi-Country Study. Patient Prefer. Adherence.

