# Supplementary material for: Real-World Data from a Multi-Center Study: Insights to Psoriatic Arthritis Care
Source: J Clin Med. 2021 Sep 11;10(18):4106. doi: 10.3390/jcm10184106 (PMC8465653; doi:10.3390/jcm10184106)
Supplement: Supplementary file 1 [file jcm-10-04106-s001.zip › jcm-1345007-supplementary.pdf]

Supplementary Material

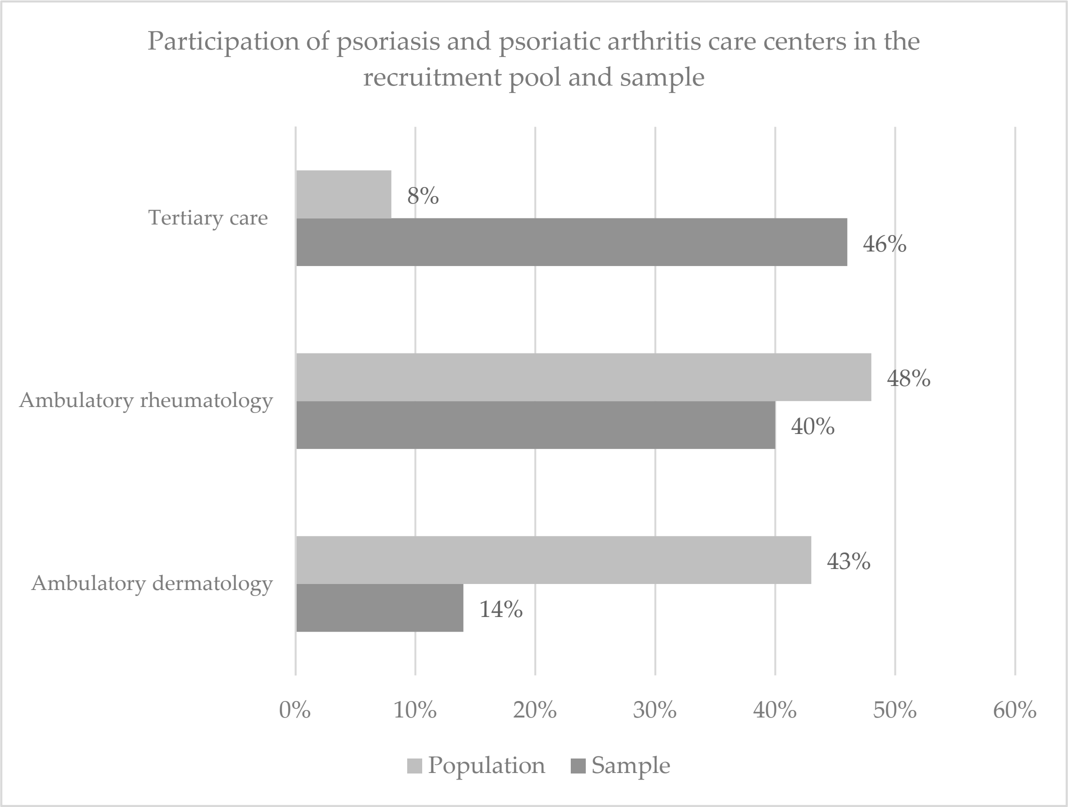

Figure S1 Representation of centers recruited in the sample and the respective proportions compared to the center recruitment pool. Due to the disparities, center-level data had to weighed to the population average.

Section 1.1. Barriers to biologic treatment based on physician responses (n=72) *(percentages refer to the proportion of providers that checked the respective questionnaire box)*.

General inclusion criteria for the drug program (65% of responders), specifically the stringent requirement of prior failure of treatments (64%), actual availability of biological drugs (43%), patient fears over side effects (39%), incomplete medical records (i.e. failure of csDMARDs has to be documented in medical records and cannot be declarative in Poland) (38%), contraindications (31%), high costs of treatment (29%), views of other providers regarding bDMARDs (26%), wrongful understanding of bDMARD therapy (24%), costs of additional testing that has to be covered by the patient (21%), assistance from others and additional costs to receive treatment in a tertiary center (17% each), patient lifestyle (10%), others (11%) and difficult to state (3%).

Section 1.2 Descriptive characteristics of group D subjects

Group D subjects were, by definition, patients with no history of biological treatment, who have not achieved low disease activity (treatment target) until present day. They are judged by rheumatology or dermatology specialists as eligible for biologics based on clinical judgment but do not fulfill local drug program criteria.

Only 3% of these patients are not aware of biologic therapy, though 8% were not informed by providers about biologics as a successive line of treatment (i.e. they were informed about biologics from other sources). 87% of patients have spoken with their physician regarding drug program procedures and in two-thirds of cases this conversation was initiated by the physician. 82% of patients were informed that they do not satisfy criteria for reimbursement (59% - unfulfilling specific criteria, 23% - active infections). The remainder of the cases were referred to a specialized center or the physicians initiated the procedure themselves. Interestingly, most patients expressed the view that they would prefer their physicians to provide information regarding biologic therapy (87%), as opposed to a trusted website (32%) or written pamphlet (26%).
